# Late-life longitudinal blood pressure trajectories as predictor of dementia

**DOI:** 10.1038/s41598-022-05680-3

**Published:** 2022-01-31

**Authors:** Allen T. C. Lee, Ada W. T. Fung, Marcus Richards, Wai C. Chan, Helen F. K. Chiu, Ruby S. Y. Lee, Linda C. W. Lam

**Affiliations:** 1 Department of Psychiatry, Faculty of Medicine, The Chinese University of Hong Kong, Hong Kong SAR, China; 2 Department of Applied Social Sciences, The Hong Kong Polytechnic University, Hong Kong SAR, China; 3 MRC Unit for Lifelong Health and Ageing at UCL, London, UK; 4 Department of Psychiatry, Faculty of Medicine, The University of Hong Kong, Hong Kong SAR, China; 5 Elderly Health Service, Department of Health, The Government of Hong Kong SAR, Hong Kong SAR, China

**Keywords:** Cognitive ageing, Dementia

## Abstract

While hypertension is widely recognized as a risk factor for dementia, few observational studies and clinical trials fully accounted for the effect of age on blood pressure (BP) changes prior to dementia onset. In this territory-wide population-based longitudinal study of 16,591 community-living dementia-free older adults, we followed their BP and cognitive status and tested if loss of longitudinal increase in BP in late life was associated with higher dementia risk in 6 years, with consideration of the confounding effects of hypertension, hypotension, BP variability, and other health problems and behaviours and, in the data analysis, exclusion of individuals who developed dementia within 3 years after baseline to minimize risk of reverse causality. Over 72,997 person-years of follow-up, 1429 participants developed dementia. We found that loss of longitudinal increase in systolic BP (defined as SBP increased by either < 10 mmHg or 10%) from baseline to Year 3 was independently associated with higher risk of incident dementia at Years 4 to 6 (adjusted OR 1.22, 95% CI 1.02–1.45, *p* = 0.03; adjusted OR 1.24, 95% CI 1.03–1.50, *p* = 0.02; respectively). Our findings suggest that late-life SBP trajectory changes might independently predict dementia onset and highlight the importance of including longitudinal BP monitoring in dementia risk assessment.

## Introduction

Hypertension has a strong but complex association with dementia^1^. While mounting evidence from longitudinal epidemiological studies suggests that hypertension increases risk of dementia, the target blood pressure (BP) for a cognitively beneficial effect remains unclear^2^. Recently, emerging evidence suggest that intensive BP lowering reduces risk of mild cognitive impairment (MCI) and dementia^3^. Nevertheless, its benefit is modest compared to the risk reduction for stroke, and importantly, hypotension and greater BP variability are associated with high risk of dementia^4,5^. Additionally, previous clinical trials have not fully accounted for the effect of age and dementia on BP changes—in mid-life, systolic and diastolic BP (SBP and DBP) increase in parallel; in late life, SBP continues to increase while DBP levels off or even declines due to arterial stiffness; and in dementia, both SBP and DBP decrease with time^6^. Indeed, higher BP has been observed to be associated with better cognition in the oldest-old^7^. More comprehensive studies are thus much needed to unravel how BP is associated with the development of dementia in older adults.

To address this knowledge gap, we conducted a large-scale longitudinal territory-wide population-based study and examined the risk of incident dementia across different SBP and DBP trajectories in late life, while taking into consideration the effects of hypertension, hypotension, BP variability, and other potential confounding factors including sociodemographics (age, sex, educational level, socioeconomic status), health problems (diabetes, hypercholesterolemia, obesity, heart diseases, sensory impairments, and depression) and lifestyle behaviours (physical exercise, intellectual and social activities, diet, smoking, and alcohol consumption), which themselves have already been shown to increase the risk of dementia, and excluding those who developed dementia shortly after baseline to reduce the risk of reverse causality. We hypothesized that loss of longitudinal increase in BP in late life was associated with higher risk of incident dementia, independent of the above potential confounding effects. Our findings might support and extend the existing literature that late-life BP changes are potential biomarker or predictor of dementia, and highlight the importance of including longitudinal BP monitoring in dementia risk assessment.

## Results

### Dementia incidence

A total of 18,298 individuals were registered. Of them, 16,591 (90.7%) were included (Supplementary Fig. 1). Their mean age was 74.5 years (SD = 4.9 years), and their mean Mini-Mental State Examination (MMSE) total score was 26.2 (SD = 3.2). They had a median follow-up period of 5.0 years, contributing to 72,997 person-years. 1429 (8.6%) had incident dementia in 6 years. They were predominantly female and older, with lower educational attainment, more co-morbidities such as hypertension, diabetes, heart diseases, sensory impairments, and depression, and less healthy lifestyle practices (Table 1). The trajectories of their MMSE total score were illustrated in Supplementary Fig. 2.

**Table 1:** Comparison of baseline characteristics between older adults with and without incident dementia in 6 years.

Characteristic	Incident dementia		*P* value
	No (*n* = 15,162)	Yes (*n* = 1429)	
Age, years	74.3 ± 4.8	76.4 ± 5.2	< 0.001^†^
Female	9525 (62.8)	1038 (72.6)	< 0.001^‡^
No schooling received	3872 (25.5)	534 (37.4)	< 0.001^‡^
Low socioeconomic class	1914 (12.6)	208 (14.6)	0.04^‡^
Hypertension	9695 (63.9)	991 (69.3)	< 0.001^‡^
Diabetes	2255 (14.9)	261 (18.3)	0.001^‡^
Hypercholesterolemia	6355 (41.9)	612 (42.8)	0.50^‡^
Obesity	5739 (37.9)	561 (39.3)	0.31^‡^
Heart diseases	1693 (11.2)	201 (14.1)	0.001^‡^
Visual impairment	4899 (32.3)	632 (44.2)	< 0.001^‡^
Hearing impairment	3417 (22.5)	365 (25.5)	0.01^‡^
Poor balance	3296 (21.7)	492 (34.4)	< 0.001^‡^
Depression	581 (3.8)	84 (5.9)	< 0.001^‡^
Physical exercise	7194 (50.6)	549 (40.8)	< 0.001^‡^
Intellectual activities	10,142 (66.9)	723 (50.6)	< 0.001^‡^
Social activities	11,594 (76.5)	1114 (78.0)	0.20^‡^
Adequate fruit and vegetable intake	7400 (48.8)	643 (45.0)	0.006^‡^
Smoking	790 (5.2)	68 (4.8)	0.46^‡^
Alcohol use	629 (4.1)	48 (3.4)	0.15^‡^
Systolic blood pressure (SBP), mmHg	141.6 ± 20.9	142.9 ± 20.8	0.02^†^
Diastolic blood pressure (DBP), mmHg	71.4 ± 10.3	70.6 ± 10.7	0.005^†^
SBP ≥ 140 mmHg	7851 (51.8)	790 (55.3)	0.01^‡^
DBP ≥ 90 mmHg	649 (4.3)	55 (3.8)	0.44^‡^
SBP ≥ 130 mmHg	11,021 (72.7)	1067 (74.7)	0.11^‡^
DBP ≥ 80 mmHg	3196 (21.1)	275 (19.2)	0.10^‡^
SBP < 100 mmHg	209 (1.4)	18 (1.3)	0.71^‡^
DBP < 60 mmHg	1742 (11.5)	197 (13.8)	0.01^‡^

Values are mean ± standard deviation for continuous variables as determined by the independent t-test^†^, and *n* (%) for categorical variables as determined by the χ2 test^‡^.

### Baseline BP and risk of incident dementia

The incident dementia group had higher SBP and lower DBP at baseline than the cognitively stable group (Table 1), but their associations were no longer significant after controlling for confounding factors (Table 2). Also, no significant association was found between systolic or diastolic hypertension (≥ 140 mmHg/90 mmHg) or hypotension (< 100 mmHg/60 mmHg) at baseline and higher risk of incident dementia after controlling for confounding factors (Tables 1 and 2). Results remained similar when the latest recommended cutoff for hypertension (SBP ≥ 130 mmHg and DBP ≥ 80 mmHg) was used in our analysis (Tables 1 and 2).

**Table 2:** Estimated odds ratios (ORs) and 95% confidence intervals (95% CI) of baseline systolic and diastolic blood pressure (SBP and DBP, respectively) for incident dementia at Years 4 to 6.

Baseline BP	Model 1		Model 2	
	OR (95% CI)	*P* value	OR (95% CI)	*P* value
Mean SBP	1.01 (1.00–1.01)	0.009	1.00 (0.99–1.01)	0.48
Mean DBP	0.99 (0.99–1.01)	0.07	0.99 (0.99–1.01)	0.58
SBP ≥ 140 mmHg^†^	1.21 (1.05–1.40)	0.01	1.10 (0.92–1.31)	0.31
DBP ≥ 90 mmHg^†^	0.99 (0.68–1.42)	0.93	1.06 (0.72–1.55)	0.78
SBP ≥ 130 mmHg^‡^	1.19 (1.00–1.40)	< 0.05	1.04 (0.86–1.26)	0.67
DBP ≥ 80 mmHg^‡^	0.91 (0.76–1.10)	0.34	0.98 (0.80–1.19)	0.83
SBP < 100 mmHg^†^	0.93 (0.47–1.84)	0.83	0.87 (0.42–1.81)	0.71
DBP < 60 mmHg^†^	1.16 (0.94–1.44)	0.17	1.06 (0.84–1.34)	0.61

Comparison was made with reference to stable SBP or DBP defined as 100–140 mmHg or 60–90 mmHg, respectively,^†^ or to stable SBP or DBP defined as 100–130 mmHg or 60–80 mmHg, respectively.^‡^ Model 1 was unadjusted, whereas Model 2 was adjusted for age, sex, educational level, socioeconomic status, hypertension, diabetes, hypercholesterolemia, obesity, heart diseases, visual impairment, hearing impairment, poor balance, depression, physical exercise, intellectual activities, social activities, fruit and vegetable consumption, smoking, and alcohol use.

### Longitudinal BP trend and risk of incident dementia

A steeper decline in SBP from baseline to Year 3 was found in participants who developed incident dementia at Years 4 to 6 (incident dementia group: 143.0 mmHg [20.9 mmHg] at baseline vs 141.4 mmHg [20.8 mmHg] at Year 3, *p* = 0.04; cognitively stable group: 141.0 mmHg [20.5 mmHg] at baseline vs 140.4 mmHg [20.3 mmHg] at Year 3, *p* = 0.001; between group difference: *p* = 0.007; Supplementary Fig. 3), whereas the decline in DBP was similar between the two groups (incident dementia group: 70.7 mmHg [10.6 mmHg] at baseline vs 69.9 mmHg [9.8 mmHg] at Year 3, *p* = 0.02; cognitively stable group: 71.4 mmHg [10.2 mmHg] at baseline vs 70.5 mmHg [10.0 mmHg] at Year 3, *p* < 0.001; between group difference: *p* = 0.09; Supplementary Fig. 4).

Persistent systolic hypertension, but not persistent diastolic hypertension or persistent hypotension, was found to be more prevalent in the incident dementia group than in the cognitively stable group, regardless of the cutoff for hypertension (Supplementary Table 1). However, its association with higher risk of incident dementia was not significant after adjusting for potential confounding factors (Table 3).

**Table 3:** Estimated odds ratios (ORs) and 95% confidence intervals (95% CI) of persistent systolic and diastolic hypertension and hypotension from baseline to Year 3 for incident dementia at Years 4 to 6.

Longitudinal BP	Model 1		Model 2	
	OR (95% CI)	*P* value	OR (95% CI)	*P* value
Persistent SBP ≥ 140 mmHg^†^	1.25 (1.05–1.50)	0.01	1.08 (0.86–1.35)	0.51
Persistent DBP ≥ 90 mmHg^†^	1.18 (0.54–2.56)	0.68	1.47 (0.66–3.25)	0.34
Persistent SBP ≥ 130 mmHg^‡^	1.19 (0.95–1.49)	0.13	1.01 (0.78–1.31)	0.96
Persistent DBP ≥ 80 mmHg^‡^	0.93 (0.72–1.20)	0.58	1.08 (0.82–1.42)	0.61
Persistent SBP < 100 mmHg^†^	0.49 (0.07–3.60)	0.48	0.59 (0.08–4.45)	0.61
Persistent DBP < 60 mmHg^†^	1.21 (0.89–1.65)	0.23	1.08 (0.77–1.52)	0.66

Comparison was made with reference to stable SBP or DBP defined as 100–140 mmHg or 60–90 mmHg, respectively,^†^ or to stable SBP or DBP defined as 100–130 mmHg or 60–80 mmHg, respectively.^‡^ Model 1 was unadjusted, whereas Model 2 was adjusted for age, sex, educational level, socioeconomic status, hypertension, diabetes, hypercholesterolemia, obesity, heart diseases, visual impairment, hearing impairment, poor balance, depression, physical exercise, intellectual activities, social activities, fruit and vegetable consumption, smoking, and alcohol use.

Variability of BP levels in the first 3 years, as reflected by the mean, coefficient of variation (CV), and adjusted standard deviation (SD), was greater in the incident dementia group (Supplementary Table 2). However, their associations with higher risk of incident dementia were not significant after adjusting for confounders (Table 4).

**Table 4:** Estimated odds ratios (ORs) and 95% confidence intervals (95% CI) of variability of systolic and diastolic blood pressure (SBP and DBP, respectively) for incident dementia at Years 4 to 6.

BP variability	Model 1		Model 2	
	OR (95% CI)	*P* value	OR (95% CI)	*P* value
SBP mean	1.01 (1.00–1.01)	0.004	1.00 (0.99–1.007)	0.41
SBP CV	1.02 (1.01–1.04)	0.004	1.01 (0.99–1.025)	0.19
SBP adjusted SD	1.01 (1.00–1.03)	0.01	1.00 (0.99–1.016)	0.45
DBP mean	0.99 (0.98–1.00)	0.05	1.00 (0.99–1.008)	0.75
DBP CV	1.02 (1.01–1.04)	0.005	1.01 (0.99–1.026)	0.15
DBP adjusted SD	1.02 (0.99–1.04)	0.1	1.01 (0.98–1.033)	0.54

Model 1 was unadjusted, whereas Model 2 was adjusted for age, sex, educational level, socioeconomic status, hypertension, diabetes, hypercholesterolemia, obesity, heart diseases, visual impairment, hearing impairment, poor balance, depression, physical exercise, intellectual activities, social activities, fruit and vegetable consumption, smoking, and alcohol use. *CV*: coefficient of variation; *SD*: standard deviation.

Loss of longitudinal increase in SBP from baseline to Year 3 was more prevalent in participants who developed incident dementia at Years 4 to 6 than those who remained cognitively stable (Supplementary Table 3). Participants whose SBP increased by < 10 mmHg or < 10% over the first 3 years were at greater risk of developing dementia in the following 3 years than those whose SBP maintained to increase by ≥ 10 mmHg or ≥ 10%, even when controlling for all other potential confounding factors (Table 5). No association was found between loss of longitudinal increase in DBP and higher risk of incident dementia.

**Table 5:** Estimated odds ratios (ORs) and 95% confidence intervals (95% CI) of loss of longitudinal blood pressure (BP) increase over the first 3 years for incident dementia at Years 4 to 6.

	Model 1		Model 2	
	OR (95% CI)	*P* value	OR (95% CI)	*P* value
SBP increased by < 10 mmHg	1.21 (1.03–1.43)	0.02	1.22 (1.02–1.45)	0.03
SBP increased by < 10%	1.23 (1.03–1.47)	0.03	1.24 (1.03–1.50)	0.02
DBP increased by < 10 mmHg	1.05 (0.84–1.32)	0.66	1.09 (0.86–1.38)	0.47
DBP increased by < 10%	1.15 (0.96–1.39)	0.13	1.19 (0.98–1.45)	0.08

Model 1 was unadjusted, whereas Model 2 was adjusted for age, sex, educational level, socioeconomic status, history of hypertension, diabetes, hypercholesterolemia, obesity, heart diseases, visual impairment, hearing impairment, poor balance, depression, physical exercise, intellectual activities, social activities, fruit and vegetable consumption, smoking, alcohol use, hypertension, hypotension, and blood pressure variability.

## Discussion

To our knowledge, no prior study has examined the longitudinal association between late-life BP trajectories and risk of dementia in community-living older adults while considering the confounding effects of hypertension, hypotension, BP variability, other health problems and behaviours, and reverse causality altogether. In this large community cohort study, we found a decline in SBP prior to dementia onset; participants with loss of longitudinal increase in SBP were over 20% more likely to develop dementia in subsequent years. Importantly, this association was present in SBP only and was not fully explained by other BP abnormalities, depression, physical health, lifestyle, and sociodemographic factors. From a clinical perspective, our findings highlight the potential role of late-life SBP trajectory as a biomarker or predictor of dementia and underscore the importance of including longitudinal BP monitoring in dementia risk assessment.

### Comparison with previous studies

Consistent with the prior literature^4,5^, we found higher SBP and greater BP variability before dementia onset. However, these BP parameters are not clinically useful in identifying people at risk of dementia because their absolute difference between those with and without incident dementia is small. While persistent hypertension and greater BP variability in late life were more prevalent in the incident dementia group than in the cognitively stable group, their associations with higher risk of dementia incidence were no longer robust after adjusting for other factors. Our findings suggest that SBP trajectory may serve as a better predictor of dementia in late life than the other BP parameters such as single BP measurement or BP variability. Our observations also appear to be in line with the current understanding that hypertension in mid-life rather than late-life might play a more important role in the development of dementia^8,9^.

Earlier observations from life-course epidemiological studies show that BP changes with age, with SBP increasing since mid-life, but decreasing in dementia^10–12^. In this study, we also found a significant difference in the SBP trajectory between the incident dementia and cognitively stable groups. Compared to participants who remained cognitively stable, those with incipient dementia showed a steep decline in SBP in late life. Additionally, we found that loss of longitudinal increase in SBP in late life was associated with higher risk of incident dementia, independent of other BP abnormalities and various confounding factors such as diabetes, heart diseases and depression which were also found to be more prevalent in our incident dementia group. Not only do our findings add to the existing literature of the longitudinal course of BP prior to clinical dementia, but they also provide additional evidence supporting the possibility of SBP trajectory being a potential predictor of dementia.

One possibility for the loss of longitudinal increase in SBP associating with higher dementia incidence is that people with impending dementia might have already started to have some cognitive impairment, thus taking drugs erratically or overdosing anti-hypertensive medication repeatedly and resulting in BP drop. However, this confounding effect of poor drug adherence alone does not appear to fully account for the observed association, which remained robust after controlling for BP variability. While the precise mechanisms have yet to be elucidated, we speculate that the dementia pathologies might disrupt autonomic regulation and cause a decline in SBP over time. Previous studies suggest that younger-old adults with atherosclerosis but not yet cognitively impaired might be at the stage when compensatory mechanisms are still functioning, so their cerebral autoregulation remains relatively effective in maintaining optimal cerebral perfusion^13,14^. Once the compensatory mechanisms are exhausted, hypoperfusion may trigger ischemia and manifestation of dementia symptoms, resulting in a vicious cycle^15–18^. These might explain the lack of benefits of intensive BP lowering in older adults with low baseline cognitive function in the SPRINT MIND study^19^ and the observation of better cognition with higher BP in the oldest-old^7^. Further investigation of the role of SBP and the effect of autonomic dysregulation on dementia will provide us with a better understanding of the underlying pathophysiological mechanisms.

While recent randomized controlled trial suggests more stringent BP control lowers risk of MCI and dementia^20^, our findings highlight the concern of intensifying BP lowering in older adults whose BP is already in a downward trend. Other factors of BP control—how quickly intensive BP control is attained, how much BP lowering is expected, and which anti-hypertensive drug is used—are likely essential in determining the long-term cognitive benefits. Examination of what the optimal BP target for older adults should be, and how it should be achieved, so that the risk of hypertension complications can be minimized without adversely affecting cognition would be much needed.

### Limitations and strengths of this study

This study had several limitations. First, given the nature of our study design, care needs to be taken when inferring a causal relationship between BP changes and dementia risk. Second, the possibility of reverse causality, though minimized in this study, cannot be completely excluded due to the potential confounding problems of people with suboptimal BP control owing to subtle cognitive problems albeit they were screened negative for significant cognitive impairment. As the development of dementia may span over a decade, our study period remains relatively short. A much longer follow-up duration, which we are currently conducting, might provide us with more robust evidence of the longitudinal association between BP changes and risk of dementia. Third, the onset and duration of hypertension and, importantly, the treatment, including the type, dosage and compliance of anti-hypertensive medication, were not assessed, though some studies found SBP decline in incipient dementia regardless of antihypertensive treatment^11,21^. Also, due to logistic limitations, BP was measured annually only, the etiology of dementia was not identified, and genotyping and neuroimaging were not performed albeit a possible role of Apolipoprotein E4 in cerebrovascular risks and a potential effect of BP on dementia pathologies in the brain^22,23^. Fourth, as some subgroups were small, the low statistical power might limit us to identify their association with dementia risk. Last, direct application of our findings to older populations with more severe hypertension and of other ethnicities requires caution, as our cohort was relatively healthy and ethnic Chinese.

Nevertheless, this study had several strengths. We followed this large territory-wide population-based cohort for a long time, with steps taken to minimize the attrition rate. Dementia was identified from comprehensive clinical assessment instead of hospital records. Various health problems and behaviours, in addition to hypotension and BP variability, were controlled in the analyses.

## Conclusions

Our findings support and extend the previous literature that BP is an important factor in the understanding of dementia risk in older adults. With changes of BP trajectory in late life being a potential biomarker or predictor of dementia, our findings suggest that not only do older adults with unintentional progressive decline in SBP warrant further dementia risk assessment, but healthcare professionals also need to be mindful in intensifying BP control in these individuals as further drop in SBP might risk hypotension and potentially accelerate their progression to clinical dementia. In future, trials should be conducted to test whether achieving optimal BP and maintaining optimal cerebral perfusion could help slow or prevent dementia onset in older populations.

## Methods

### Study design, setting, and participants

This longitudinal observational study was conducted at all 18 Elderly Health Centres (EHCs) of the Department of Health of the Government of Hong Kong, which provide regular physical and cognitive health assessments to local older adults. Inclusion criteria for this study were aged 65 and older, Chinese ethnicity, living in the community, and free of dementia at baseline. Exclusion criteria were living in nursing homes; having stroke, Parkinson’s disease, or clinical dementia; scoring below the education-specific cutoff on the Cantonese version of the Mini-Mental State Examination (MMSE)^24^, or not completing cognitive or BP assessment at baseline. Participants were followed up at the EHCs for 6 years to the outcome of incident dementia. During the study period, participants received annual standardized clinical assessment of cognitive status and BP, together with comprehensive examination of a wide range of physical and psychiatric comorbidities and lifestyle behaviours. To minimize loss to follow-up, those who missed the follow-up assessments were actively traced and interviewed by geriatric psychiatrists at EHCs or at home; the names of those not traceable were verified with the Deaths Registry for the cause of death, including dementia. In this study, informed consent was obtained from all participants, or from their relatives if they were mentally incapable of giving consent before the follow-up assessment was conducted. This study was approved by the Ethics Committee of the Department of Health of the Government of Hong Kong and the Joint Clinical Research Ethics Committee of the Chinese University of Hong Kong and the New Territories East Cluster of the Hospital Authority. All methods were carried out in accordance with relevant guidelines and regulations.

### Assessment of BP control

At baseline and follow-up, brachial BP was measured with an electronic sphygmomanometer using an appropriately sized cuff after participants sat at rest for at least 5 min. Two readings were taken in at least 5 min apart for participants whose first BP reading was ≥ 140/90 mmHg. The average of the two readings were used for analysis.

BP variability was examined using intra-individual mean (BP-MEAN), standard deviation (BP-SD), coefficient of variation (BP-CV), and adjusted standard deviation (adjusted-BP-SD) of BP. BP-CV, defined as the ratio of BP-SD to BP-MEAN, serves as a better marker for BP variability because it corrects for larger SDs due to higher absolute values of BP-MEAN, whereas adjusted-BP-SD reflects the inter-individual differences after correcting for the number of BP assessments.

### Assessment of dementia cases

The outcome of this study was incident dementia in 6 years. At baseline and follow-up, participants underwent comprehensive cognitive assessments by the EHC physicians, including a detailed history, the Abbreviated Mental Test, and the MMSE. Participants who missed these but agreed to a follow-up interview received the clinical examination, MMSE, and Clinical Dementia Rating (CDR) by geriatric psychiatrists. Dementia was diagnosed according to the *International Statistical Classification of Diseases and Related Health Problems, Tenth Revision* (ICD-10) and a CDR of 1 to 3^25^. A panel of geriatric psychiatrists reviewed all the diagnosis of dementia independently. For cases whose diagnosis was uncertain or in disagreement, the principal investigator adjudicated the final diagnosis.

### Assessment of other variables

Sociodemographic factors (age, sex, educational level, and socioeconomic status), physical and psychiatric comorbidities (hypertension, type 2 diabetes mellitus, hypercholesterolemia, obesity, heart diseases, visual and hearing impairments, poor balance, and depression), and lifestyle behaviours (physical, intellectual, and social activities, fruit and vegetable intake, smoking, and alcohol use) were examined. Low socioeconomic status was defined as receiving social security from the government. All medical diseases were diagnosed by physicians according to the ICD-10. Obesity was defined as body mass index equal to or greater than 25 kg/m^2^ according to the Asian references^26^. Hearing impairment was defined as 1- and 2-kHz loss of ≥ 40 decibels in the better ear during audiometric testing (Audioscope, Welch Allyn). Visual impairment was defined as ≥ 0.48 LogMAR^27^. Poor balance was defined as failing the single-leg balance test^28^. A standardized self-reported questionnaire based on locally validated leisure activity classification was used to assess lifestyle behaviours in the prior month^29^; regular physical (aerobic and mind–body exercises), intellectual (reading books, newspapers, or magazines; playing board games, Mahjong or card games; and betting on horse racing), and social activities (joining a social centre, participating in voluntary work, meeting relatives or friends, and attending religious activities), adequate fruit and vegetable intake (consuming at least 3 servings of vegetables and 2 servings of fruits a day), current smoking, and alcohol use were defined as previously reported^30–32^.

### Sample size estimation

Sample size estimation was performed using the Power and Precision software, version 3.0 (Biostat; https://www.power-analysis.com/software_overview.htm). Sample size was calculated based on estimates of incidence rate of dementia (6% in 6 years) and people with hypertension having an odds ratio of 2.0 for dementia from previous literature^10^. With alpha set at 0.05, a baseline sample of 10,000 participants would yield at least 80% power for detection of dementia at follow-up.

### Statistical analysis

Statistical analysis was performed using IBM SPSS Statistics, version 26.0 (IBM Corp; https://www.ibm.com/analytics/spss-statistics-software). Baseline continuous and categorical variables were compared between participants with and without incident dementia in 6 years by independent t-test and Chi-squared (*χ*^2^) test, respectively. The level of statistical significance was set at *P* < 0.05 (2-tailed). With reference to the Seventh Report of the Joint National Committee on Prevention, Detection, Evaluation, and Treatment of High Blood Pressure (JNC 7), systolic and diastolic hypertension were defined as ≥ 140 mmHg and ≥ 90 mmHg, respectively, and hypotension as < 100 mmHg and < 60mmHg^33^. Additionally, to test if the risk of incident dementia was different with the new BP cutoff for hypertension according to the latest American College of Cardiology/American Heart Association Hypertension Guideline^34^, the analyses were repeated using ≥ 130 mmHg and ≥ 80 mmHg as systolic and diastolic hypertension, respectively. Multivariable logistic regression analysis was performed to test if hypertension and hypotension at baseline were associated with higher risk of incident dementia with reference to stable BP (SBP of 100–140 mmHg [or 100–130 mmHg based on the latest cutoff]; DBP of 60–90 mmHg [or 60–80 mmHg based on the latest cutoff]), even after adjusting for sociodemographic factors (age, sex, educational level, socioeconomic class), physical and psychiatric comorbidities (history of hypertension, type 2 diabetes mellitus, hypercholesterolemia, obesity, heart diseases, visual impairment, hearing impairment, poor balance, and depression), and lifestyle behaviours (physical exercise, intellectual activities, social activities, adequate fruit and vegetable intake, smoking, alcohol use) and excluding those who developed dementia within 3 years after baseline. Model 1 was unadjusted, whereas Model 2 was adjusted for all the potential confounding factors as described above. The odds ratios (ORs) were computed to yield point estimates with 95% confidence intervals (95% CIs).

To examine if longitudinal trend of BP was different between the cognitively stable and incident dementia groups, the BP changes from baseline to Year 3 were compared between participants with and without incident dementia at Years 4 to 6 using General Linear Model repeated measures, with the last observation of BP readings carried forward for those who missed the follow-up BP assessment, and the Bonferroni adjustment applied for multiple comparison. To test if persistent hypertension and hypotension were associated with higher risk of incident dementia, the proportion of participants whose BP was persistently equal to or greater than 140/90 mmHg (or 130/80 mmHg based on the latest recommendation) and less than 100/60 mmHg, respectively, over the first 3 years was compared between those with and without incident dementia in the subsequent 3 years. Additionally, to test if BP variability was associated with higher risk of dementia, the mean, CV, and adjusted SD of SBP and DBP measured from baseline to Year 3 were compared between those with and without incident dementia at Years 4 to 6; with adjusted SD already considered the number of BP measurements, last observation carried forward was not applied here. Given the possible effect of age and dementia on BP trajectories, we tested if the loss of longitudinal increase in BP in late life was associated with higher dementia incidence. The ORs for incident dementia in the following 3 years in participants with absolute and relative increase of BP of less than 10 mmHg and 10%, respectively, over the first 3 years were estimated using the regression analyses, with hypertension, hypotension, BP variability, and other confounders including demographics, health problems, and lifestyle behaviours controlled for.

### Ethical approval

This study was approved by the Ethics Committee of the Department of Health of the Government of Hong Kong and the Joint Clinical Research Ethics Committee of the Chinese University of Hong Kong and the New Territories East Cluster of the Hospital Authority.

## Supplementary Information

Supplementary Information.

## Data Availability

The data that support the findings of this study are available from the corresponding author upon reasonable request.
