# Supplementary material for: Late-life longitudinal blood pressure trajectories as predictor of dementia
Source: Sci Rep. 2022 Jan 31;12:1630. doi: 10.1038/s41598-022-05680-3 (PMC8803958; doi:10.1038/s41598-022-05680-3)
Supplement: Supplementary file 1 — Supplementary Information. [file 41598_2022_5680_MOESM1_ESM.docx]

This supplementary file has 4 supplementary figures and 3 supplementary tables.

**Supplementary Figures**

**
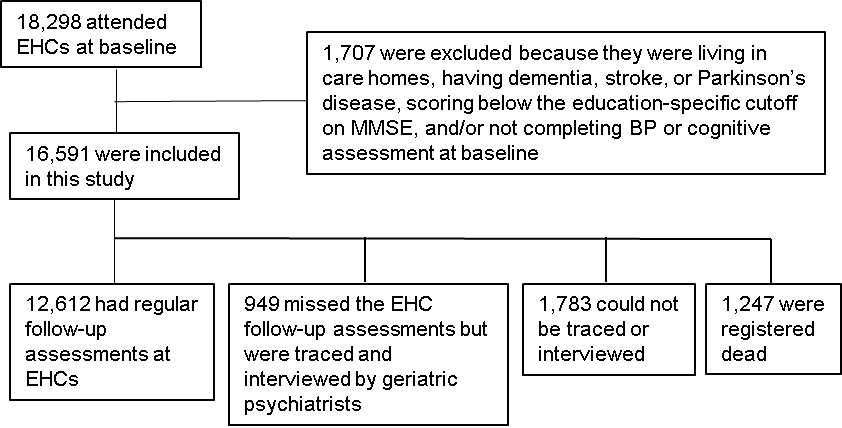
**

Supplementary Figure 1. Flow chart of the participants in this study.


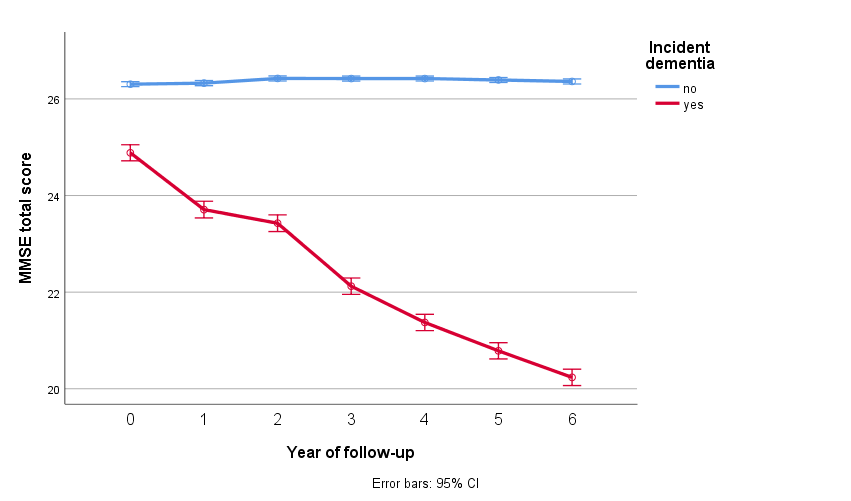


Supplementary Figure 2. Differences in trajectories of MMSE total score between participants with and without incident dementia.


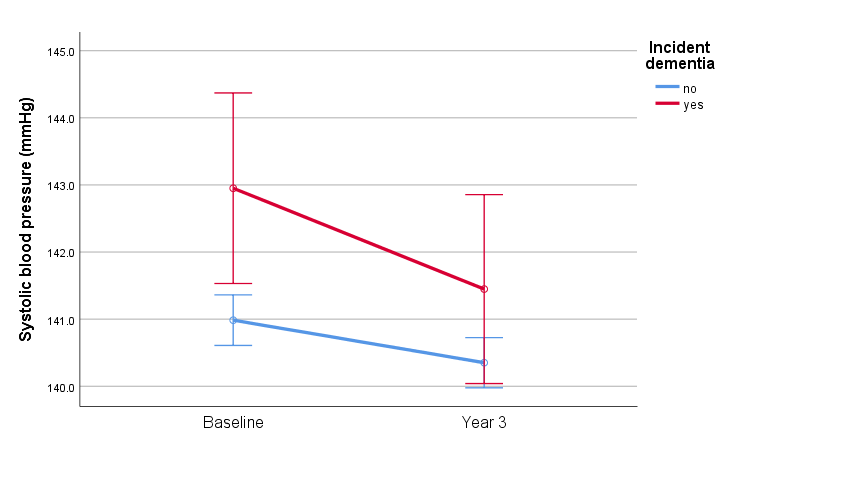


Supplementary Figure 3. Differences in changes of systolic blood pressure from baseline to Year 3 between participants with and without incident dementia from Years 4 to 6.


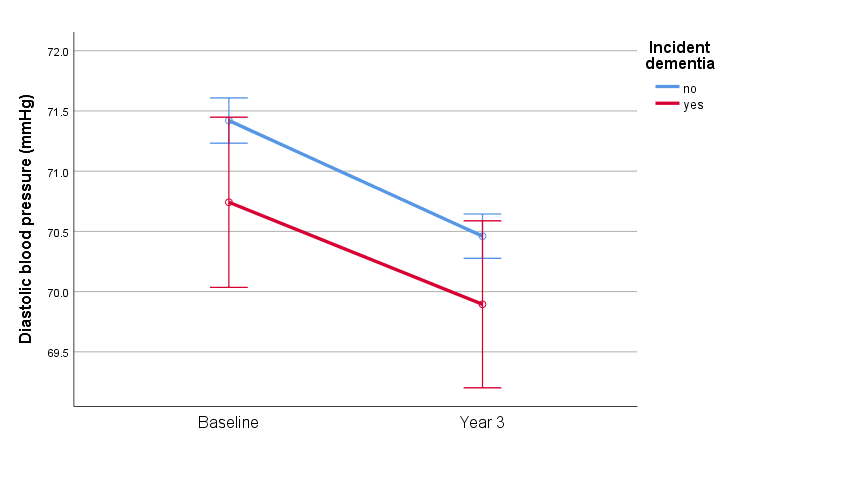


Supplementary Figure 4. Differences in changes of diastolic blood pressure from baseline to Year 3 between participants with and without incident dementia from Years 4 to 6.

**Supplementary Tables**

Supplementary Table 1. Comparison of persistent hypertension or hypotension from baseline to Year 3 between participants with and without incident dementia at Years 4 to 6. Comparison was made with reference to stable SBP or DBP defined as 100–140mmHg or 60–90mmHg, respectively,^†^ or to stable SBP or DBP defined as 100–130mmHg or 60–80mmHg, respectively.^‡^ Values are n (%) as determined by the χ^2^ test.

| Longitudinal BP | Number of participants | Incident Dementia | | *P* value |
| --- | --- | --- | --- | --- |
|  |  | No | Yes |  |
| Persistent SBP≥140mmHg^†^ | 4149 | 3842 (33.8) | 307 (38.3) | 0.01 |
| Persistent DBP≥90mmHg^†^ | 91 | 84 (0.7) | 7 (0.9) | 0.67 |
| Persistent SBP≥130mmHg^‡^ | 7119 | 6618 (58.2) | 501 (62.5) | 0.02 |
| Persistent DBP≥80mmHg^‡^ | 1095 | 1023 (9.0) | 72 (9.0) | 0.99 |
| Persistent SBP<100mmHg^†^ | 33 | 32 (0.3) | 1 (0.1) | 0.41 |
| Persistent DBP<60mmHg^†^ | 583 | 537 (4.7) | 46 (5.7) | 0.19 |

Supplementary Table 2. Comparison of variability of systolic and diastolic blood pressure (SBP and DBP, respectively) measured from baseline to Year 3 between participants with and without incident dementia at Years 4 to 6. Values are mean ± standard deviation for continuous variables as determined by the independent t-test. *CV*: coefficient of variation; *SD*: standard deviation.

| BP variability | Incident Dementia | | *P* value |
| --- | --- | --- | --- |
|  | No | Yes |  |
| SBP mean, mmHg | 140.2 ± 16.7 | 141.9 ± 17.5 | 0.004 |
| SBP CV | 8.7 ± 5.3 | 9.4 ± 6.0 | <0.05 |
| SBP adjusted SD | 10.9 ± 6.9 | 11.6 ± 7.7 | 0.01 |
| DBP mean, mmHg | 70.9 ± 8.3 | 70.3 ± 8.8 | 0.05 |
| DBP CV | 8.5 ± 5.1 | 9.1 ± 6.1 | 0.005 |
| DBP adjusted SD | 5.3 ± 3.2 | 5.5 ± 3.7 | 0.1 |

Supplementary Table 3. Comparison of loss of longitudinal blood pressure (BP) increase over the first 3 years between participants with and without incident dementia at Years 4 to 6. Values are n (%) as determined by the χ^2^ test.

|  | Number of participants | Incident Dementia | | *P* value |
| --- | --- | --- | --- | --- |
|  |  | No | Yes |  |
| SBP increased by <10mmHg | 8825 (53.2) | 8216 (72.2) | 609 (75.9) | 0.02 |
| SBP increased by <10% | 9389 (56.6) | 8745 (76.9) | 644 (80.3) | 0.03 |
| DBP increased by <10mmHg | 10720 (64.6) | 10010 (88.0) | 710 (88.5) | 0.66 |
| DBP increased by <10% | 9724 (58.6) | 9067 (79.7) | 657 (81.9) | 0.13 |
